# Viruses and Immunity in Transplant Patients

**DOI:** 10.1155/2013/492352

**Published:** 2013-11-27

**Authors:** Rossana Cavallo, Guido Antonelli, Hans H. Hirsch

**Affiliations:** ^1^University of Turin, Microbiology and Virology Unit; Azienda Ospedaliera Città della Salute e della Scienza di Torino, Via Santena 9, 10126 Turin, Italy; ^2^University of Rome “La Sapienza”, Department of Molecular Medicine, Piazzale Aldo Moro, 00185 Rome, Italy; ^3^University of Basel, Transplantation and Clinical Virology, Division Infection Diagnostics, Department Biomedicine (Haus Petersplatz), Petersplatz 10, CH-4003 Basel, Switzerland; ^4^Infectious Disease and Hospital Epidemiology, University Hospital Basel, Petersgraben 4, CH-4031 Basel, Switzerland

Viral infections significantly influence the outcome of solid organ and hematopoietic stem cell transplant recipients.

 Induction, maintenance, and intensification of immunosuppression in the context of antirejection treatment are key factors for viral replication and progression toward disease. This complex interaction of patient, virus, and graft determinants is modulated by the net balance of immunosuppression and its impact on virus-specific immune control. 

The clinical development of immunosuppressive agents is facilitated by the understanding of the mechanisms of T-cell activation, thus allowing for the identification of potential new targets and protocols. Recently developed immunosuppressive agents are able to mediate profound and prolonged immunosuppression. Their use is associated both with a reduced incidence of rejection and with an increased susceptibility to infections.

The immunologic control of viral replication in a host requires both innate and adaptive immune responses. In the past decade, virological monitoring of transplant patients has been widely established both for screening transplant patients at risk for virus replication as well as monitoring antiviral treatment responses. More recently, however, viroimmunological evaluation has been explored as a potentially useful tool for better clinical and therapeutic management. 

This special issue encompasses original research and review articles that aim to stimulate the continuing efforts to understand the interplay between host and viruses in the transplant setting. This knowledge may help to define strategies for improved diagnostics, monitoring, and therapeutic management through antiviral prophylaxis and preemptive therapy approach. The papers explore aspects of antiviral immunity, methods and significance of viroimmunological monitoring, and relation between antirejection therapies and viral reactivation in both solid organ and hematopoietic stem cell transplant recipients.

A particular role in the transplant setting is represented by persistently infecting viruses that are characterized by latency, such as herpesviruses and polyomaviruses. The members of these two families are associated with relevant complications in terms of morbidity and morbidity in transplant recipients.

Among the members of the Herpesviridae family, human cytomegalovirus (HCMV) is clearly recognized as the major opportunistic agent complicating the outcome of the graft as it may reactivate from latency sites due to antirejection immunosuppressive treatment or be responsible for primary infection in seronegative recipients. Virus-specific cellular immune response is fundamental in controlling HCMV replication in transplant patients; in fact, the recovery of HCMV-specific CD4+ and CD8+ T-cell response has been related to protection and control of viral replication and reduced rates of infections and disease, both systemic and organ-specific. In recent years, the evaluation of immunological response to HCMV has been recommended, besides virological monitoring of viral load by molecular methods, in the management of transplant recipients. As described in the International Consensus Guidelines on the Management of Cytomegalovirus in Solid Organ transplantation (Kotton et al., 2013), different methods have been developed for evaluating HCMV-specific immune response. 

In the paper by E. V. Ravkov et al., different basic parameters for validation of HCMV-specific tetramer staining and peptide stimulation assays have been examined, thus providing measures of test performance for HCMV immune competence assays for the characterization of CD8+ T-cell response; the method has been validated by evaluating the potential use of HCMV-specific CD8+ cells numbers and functional and cytolytic responses in hematopoietic stem cell transplant recipients.

In the paper by I. Gayoso et al., the phenotype and interferon-gamma production of HCMV-specific T cells have been characterized using the Quantiferon-HCVM assay in a group of hematopoietic stem cell transplant recipients, and the association with clinical variables has been studied. The authors found the occurrence of a differentiated phenotype in HCMV-specific CD8+ T-cells in recipients from older donors, whereas HCMV replication after transplantation, recipient age, and stem cell source were associated with the production of interferon-gamma in response to HCMV epitopes. 

Among herpesviruses, also Epstein-Barr virus (EBV) is associated with potentially life-threatening conditions: in particular, EBV-driven posttransplant lymphoproliferative disorders (PTLD), with early identification of aberrant EBV activity being associated with prevention of progression to B-cell lymphoma. In the study by A. E. Greijer et al., the authors evaluated EBV-DNA load and RNA profiles in plasma and cellular blood specimens from stem cell transplant recipients, solid organ transplant patients, including a subgroup of patients with chronically elevated viral load. The study evidenced that EBV-DNA was differently distributed between white cells and plasma in solid organ transplantation versus hematopoietic stem cell transplantation; EBV-RNA profiling in blood resulted in additional value in terms of understanding viral activity in patients with elevated EBV-DNA load. 

Polyomaviruses BK and JC have been increasingly recognized as a cause of transplant complication, particularly in kidney transplant recipients. BKV has been associated with a severe nephropathy potentially leading to the loss of the graft. A relevant burden of the literature has evidenced that the main determinants related to the development of nephropathy are the overall level of immunosuppression and the failure of immune surveillance in the presence of viral reactivation, with the most important role played by the cellular immune response. 

In the study by E. Girmanova and coauthors, the gene expression profiles of 90 target genes associated with immune response have been evaluated in kidney graft biopsy material by comparing three different groups, including control patients with no evidence of BK reactivation, infected asymptomatic patients, and patients with BK nephropathy. The analysis revealed several biological networks associated with BK virus immune control.

In the study by M. Cioni and coauthors, the potential role of BKV agnoprotein in immune evasion by downregulating HLA expression was explored based on the fact that it is abundantly expressed later in the viral life cycle, but specific cellular and humoral immune responses are low or absent. The study evidenced that unlike a similar protein in Herpes simplex called ICP47, the BKV agnoprotein does not contribute to viral immune evasion by downregulating HLA-ABC expression or interfering with HLA-DR expression or peptide-dependent T-cell cytotoxicity.

The role of polyomavirus JC replication and impact on graft function and survival in kidney transplantation has been reviewed in the paper by S. Delbue et al., particularly focusing on the biology of JCV and its pathological features, with a wide review of the literature on JCV in the transplant setting.

Considering liver transplant recipients, a relevant issue is represented by hepatitis C virus (HCV) in terms of both leading indication for liver transplantation and posttransplant reinfection of the graft. This has been reported to be universal and often results in accelerated progression to liver failure; moreover, treatment of recurrence after liver transplantation is often compromised by enhanced adverse effects and limited efficacy of interferon-based therapy. S. H. Hsu and coauthors present a review on the specific biological and clinical aspects regarding HCV-reinfection posttransplantation and suggest potential lines of intervention in terms of therapeutic strategies.

Besides persistently infecting viruses, also pathogens responsible of community-acquired acute infections such as respiratory viruses may account for complications in the transplant setting. For example, human rhinoviruses generally cause mild infections of the upper respiratory tract; however, in immunocompromised subjects, rhinoviruses can reach the lower airways causing serious illness. In the study by F. Canducci and coauthors, the occurrence and persistence and the pathogenic potential of human rhinoviruses (and the novel human enterovirus 109) have been retrospectively evaluated by molecular techniques and phylogenetic analysis in specimens from hematopoietic stem cell transplant adult recipients. The authors reported not only sequential infections by different rhinoviruses subtypes but also prolonged viral shedding not constantly associated with symptom.

In conclusion, viral agents may be associated with relevant morbidity and mortality in the transplant setting. The role of immune surveillance and factors influencing immune response are being extensively studied. This issue specifically reports some of the more updated researches and review considerations on the topic.

*Rossana Cavallo* *Guido Antonelli* *Hans H. Hirsch*

